# *catena*-Poly[[*trans*-bis­(cyclo­hexane-1,2-diamine-κ^2^ *N*,*N*)cadmium]-μ-iodido-(diiodidocadmium)-μ-iodido]

**DOI:** 10.1107/S1600536812024828

**Published:** 2012-06-13

**Authors:** Sheng-Feng Cui, Qin-Mei Wen, Cheng-He Zhou

**Affiliations:** a Laboratory of Bioorganic and Medicinal Chemistry, School of Chemistry and Chemical Engineering, Southwest University, Chongqing 400715, People’s Republic of China

## Abstract

In the title compound, [Cd_2_I_4_(C_6_H_14_N_2_)_2_]_*n*_, there are two independent Cd^II^ ions. One Cd^II^ ion is coordinated in a slightly distorted octa­hedral coordination environment by four N atoms from two cyclo­hexane-1,2-diamine ligands and two iodido ligands. The other Cd^II^ ion is coordinated by four iodido ligands in a slightly distorted tetra­hedral coordination environment. Two of the iodido ligands act as bridging ligands connecting Cd^II^ ions and forming a one-dimensional polymer along [010]. In the crystal, N—H⋯I hydrogen bonds connect the one-dimensional structure into a two-dimensional framework parallel to (001).

## Related literature

For general information on supra­molecular recognition, see: Zhou *et al.* (2009 ▶, 2010 ▶). For information on the selective recognition of Cd^II^ ions, see: Soisungwan (2012 ▶).
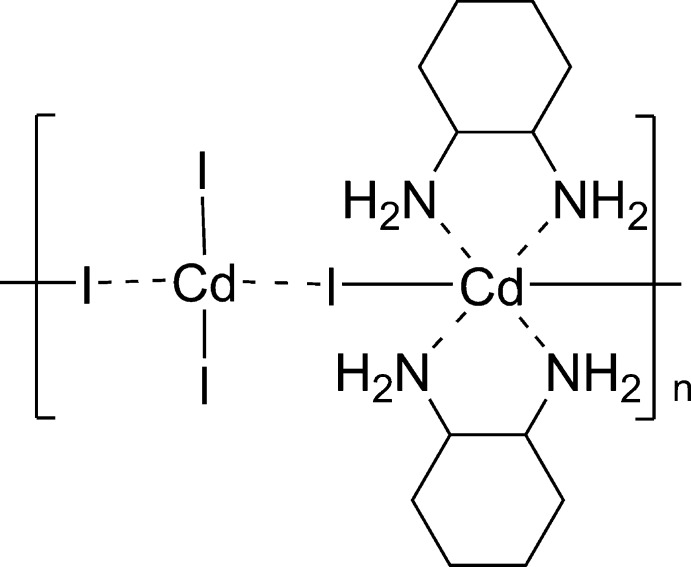


## Experimental

### 

#### Crystal data

[Cd_2_I_4_(C_6_H_14_N_2_)_2_]
*M* *_r_* = 960.78
Monoclinic, 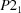

*a* = 9.6627 (5) Å
*b* = 12.1821 (7) Å
*c* = 10.9665 (6) Å
β = 109.584 (1)°
*V* = 1216.21 (12) Å^3^
*Z* = 2
Mo *K*α radiation
μ = 6.83 mm^−1^
*T* = 296 K
0.35 × 0.33 × 0.32 mm

#### Data collection

Bruker SMART APEXII CCD diffractometer
Absorption correction: multi-scan (*SADABS*; Bruker, 2009 ▶) *T*_min_ = 0.199, *T*_max_ = 0.219
9784 measured reflections
4039 independent reflections
3967 reflections with *I* > 2σ(*I*)
*R*_int_ = 0.026

#### Refinement

*R*[*F* ^2^ > 2σ(*F* ^2^)] = 0.021
*wR*(*F* ^2^) = 0.050
*S* = 1.03
4039 reflections
201 parameters
1 restraint
H-atom parameters constrained
Δρ_max_ = 0.86 e Å^−3^
Δρ_min_ = −0.62 e Å^−3^
Absolute structure: Flack (1983 ▶), 1626 Friedel pairs
Flack parameter: 0.03 (2)

### 

Data collection: *APEX2* (Bruker, 2009 ▶); cell refinement: *SAINT* (Bruker, 2009 ▶); data reduction: *SAINT*; program(s) used to solve structure: *SHELXS97* (Sheldrick, 2008 ▶); program(s) used to refine structure: *SHELXL97* (Sheldrick, 2008 ▶); molecular graphics: *SHELXTL* (Sheldrick, 2008 ▶); software used to prepare material for publication: *SHELXTL*.

## Supplementary Material

Crystal structure: contains datablock(s) global, I. DOI: 10.1107/S1600536812024828/lh5467sup1.cif

Structure factors: contains datablock(s) I. DOI: 10.1107/S1600536812024828/lh5467Isup2.hkl

Additional supplementary materials:  crystallographic information; 3D view; checkCIF report

## Figures and Tables

**Table 1:** Hydrogen-bond geometry (Å, °)

*D*—H⋯*A*	*D*—H	H⋯*A*	*D*⋯*A*	*D*—H⋯*A*
N1—H1*B*⋯I4^i^	0.90	2.84	3.723 (4)	167
N2—H2*B*⋯I4^ii^	0.90	2.81	3.681 (5)	162
N3—H3*A*⋯I3^i^	0.90	2.94	3.809 (4)	162
N4—H4*A*⋯I3^ii^	0.90	3.03	3.875 (5)	157

Symmetry codes: (i) 
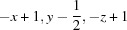
; (ii) 
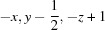
.

**Fig. 1.**
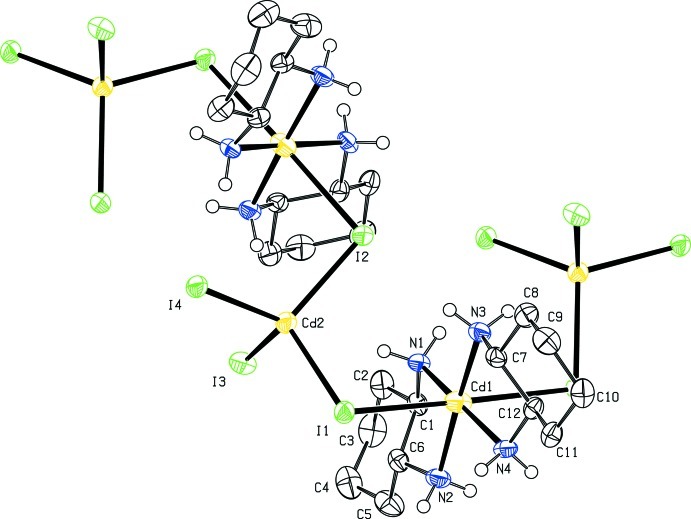
Part of the one-dimensional structure showing displacement ellipsoids at the 50% probability level. H atoms bonded to C atoms are not shown and only the asymmetric unit is labeled.

**Fig. 2.**
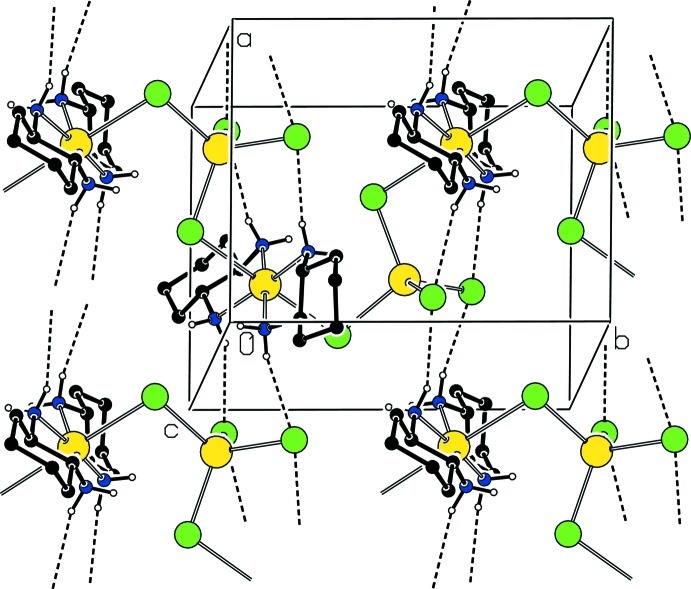
Part of the crystal structure with hydrogen bonds shown as dashed lines. H atoms bonded to C atoms are not shown.

## supplementary crystallographic information

### Comment

Organometallic supramolecular chemistry is an increasingly active interdiscipline, which is a new extensive expansion of many natural sciences including chemical, pharmaceutical, biological and material scicences. The supramolecular recognition field attracts increasingly special attention (Zhou *et al.*, 2009;2010). Our interest is to develop a novel efficient supramolecule for selective recognition of Cd^II^ ions (Soisungwan, 2012). Herein, the molecular structure of title compound is reported.

There are two independent Cd^II^ ions. One Cd^II^ ion is coordinated in a slightly distorted octahedral coordination environment by four N atoms from two cyclohexane-1,2-diamine ligands and two iodide ligands. The other Cd^II^ ion is coordinated by four iodide ligands in a slightly distorted tetrahedral coordination enviroment. Two of the iodide ligands act as bridging to connect Cd^II^ ions and form a one-dimensional polymer along [010] (Fig. 1). In the crystal, N—H···I hydrogen bonds connect the one-dimensional structure in a two-dimensional framework parallel to (001) (Fig. 2).

### Experimental

A mixture of cyclohexane-1,2-diamine (0.11 g, 0.1 mol) and cadmium (II) iodide (0.38 g, 0.1 mol) in ethanol (10.0 ml) was stirred for 8 h under reflux. The white formed precipitate was filtered and then washed with cold methanol to afford a yellow solid. A crystal suitable for X-ray analysis was grown from a solution of the title compound in chloroform by slow evaporation at room temperature.

### Refinement

H atoms were placed in calculated positions with C—H = 0.97-0.98 and N—H = 0.90Å Å. The *U*_iso_(H) values were set equal to 1.2U_eq_(C,N).

### Crystal data

**Table:**

[Cd_2_I_4_(C_6_H_14_N_2_)_2_]	*F*(000) = 872
*M**_r_* = 960.78	*D*_x_ = 2.624 Mg m^−^^3^
Monoclinic, *P*2_1_	Mo *K*α radiation, λ = 0.71073 Å
Hall symbol: P 2yb	Cell parameters from 2403 reflections
*a* = 9.6627 (5) Å	θ = 2.0–26.0°
*b* = 12.1821 (7) Å	µ = 6.83 mm^−^^1^
*c* = 10.9665 (6) Å	*T* = 296 K
β = 109.584 (1)°	Block, colorless
*V* = 1216.21 (12) Å^3^	0.35 × 0.33 × 0.32 mm
*Z* = 2	

### Data collection

**Table:**

Bruker SMART APEXII CCD diffractometer	4039 independent reflections
Radiation source: fine-focus sealed tube	3967 reflections with *I* > 2σ(*I*)
Graphite monochromator	*R*_int_ = 0.026
φ and ω scans	θ_max_ = 26.0°, θ_min_ = 2.0°
Absorption correction: multi-scan (*SADABS*; Bruker, 2009)	*h* = −11→11
*T*_min_ = 0.199, *T*_max_ = 0.219	*k* = −14→14
9784 measured reflections	*l* = −13→12

### Refinement

**Table:**

Refinement on *F*^2^	Hydrogen site location: inferred from neighbouring sites
Least-squares matrix: full	H-atom parameters constrained
*R*[*F*^2^ > 2σ(*F*^2^)] = 0.021	*w* = 1/[σ^2^(*F*_o_^2^) + (0.0249*P*)^2^ + 0.0879*P*] where *P* = (*F*_o_^2^ + 2*F*_c_^2^)/3
*wR*(*F*^2^) = 0.050	(Δ/σ)_max_ = 0.002
*S* = 1.03	Δρ_max_ = 0.86 e Å^−^^3^
4039 reflections	Δρ_min_ = −0.62 e Å^−^^3^
201 parameters	Extinction correction: *SHELXL97* (Sheldrick, 2008), Fc^*^=kFc[1+0.001xFc^2^λ^3^/sin(2θ)]^-1/4^
1 restraint	Extinction coefficient: 0.0054 (2)
Primary atom site location: structure-invariant direct methods	Absolute structure: Flack (1983), 1626 Friedel pairs
Secondary atom site location: difference Fourier map	Flack parameter: 0.03 (2)

### Special details

**Table:**

Geometry. All esds (except the esd in the dihedral angle between two l.s. planes) are estimated using the full covariance matrix. The cell esds are taken into account individually in the estimation of esds in distances, angles and torsion angles; correlations between esds in cell parameters are only used when they are defined by crystal symmetry. An approximate (isotropic) treatment of cell esds is used for estimating esds involving l.s. planes.
Refinement. Refinement of F^2^ against ALL reflections. The weighted R-factor wR and goodness of fit S are based on F^2^, conventional R-factors R are based on F, with F set to zero for negative F^2^. The threshold expression of F^2^ > 2sigma(F^2^) is used only for calculating R-factors(gt) etc. and is not relevant to the choice of reflections for refinement. R-factors based on F^2^ are statistically about twice as large as those based on F, and R- factors based on ALL data will be even larger.

### Fractional atomic coordinates and isotropic or equivalent isotropic displacement parameters (Å^2^)

**Table:**

	*x*	*y*	*z*	*U*_iso_*/*U*_eq_	
C1	0.2321 (6)	0.2128 (4)	0.2157 (5)	0.0265 (11)	
H1C	0.2580	0.1389	0.1955	0.032*	
C2	0.2491 (7)	0.2898 (5)	0.1134 (6)	0.0381 (14)	
H2C	0.3452	0.2788	0.1057	0.046*	
H2D	0.2444	0.3649	0.1412	0.046*	
C3	0.1322 (8)	0.2735 (6)	−0.0191 (6)	0.0480 (17)	
H3C	0.1428	0.3297	−0.0780	0.058*	
H3D	0.1458	0.2026	−0.0536	0.058*	
C4	−0.0207 (8)	0.2797 (6)	−0.0097 (6)	0.0482 (17)	
H4C	−0.0368	0.3518	0.0205	0.058*	
H4D	−0.0936	0.2678	−0.0943	0.058*	
C5	−0.0360 (7)	0.1927 (6)	0.0838 (6)	0.0429 (15)	
H5A	−0.0220	0.1209	0.0516	0.051*	
H5B	−0.1347	0.1955	0.0879	0.051*	
C6	0.0746 (6)	0.2081 (5)	0.2196 (5)	0.0268 (11)	
H6A	0.0539	0.2790	0.2521	0.032*	
C7	0.4059 (6)	0.1103 (4)	0.8002 (5)	0.0257 (11)	
H7A	0.3415	0.1640	0.8209	0.031*	
C8	0.5348 (7)	0.0864 (5)	0.9240 (6)	0.0352 (14)	
H8A	0.6038	0.0383	0.9032	0.042*	
H8B	0.5853	0.1546	0.9570	0.042*	
C9	0.4876 (7)	0.0330 (6)	1.0288 (6)	0.0405 (15)	
H9A	0.4309	0.0853	1.0594	0.049*	
H9B	0.5741	0.0137	1.1012	0.049*	
C10	0.3952 (7)	−0.0700 (5)	0.9800 (6)	0.0376 (14)	
H10A	0.4557	−0.1267	0.9614	0.045*	
H10B	0.3588	−0.0973	1.0467	0.045*	
C11	0.2670 (7)	−0.0446 (4)	0.8590 (6)	0.0303 (12)	
H11A	0.2011	0.0062	0.8802	0.036*	
H11B	0.2129	−0.1116	0.8268	0.036*	
C12	0.3179 (6)	0.0057 (4)	0.7529 (5)	0.0254 (11)	
H12A	0.3831	−0.0474	0.7327	0.030*	
N1	0.3315 (5)	0.2411 (4)	0.3470 (4)	0.0278 (10)	
H1A	0.3260	0.3136	0.3606	0.033*	
H1B	0.4247	0.2252	0.3537	0.033*	
N2	0.0665 (5)	0.1242 (4)	0.3116 (5)	0.0325 (11)	
H2A	0.0675	0.0570	0.2778	0.039*	
H2B	−0.0175	0.1316	0.3294	0.039*	
N3	0.4548 (5)	0.1568 (4)	0.6965 (4)	0.0283 (10)	
H3A	0.5339	0.1198	0.6928	0.034*	
H3B	0.4804	0.2276	0.7141	0.034*	
N4	0.1941 (5)	0.0265 (4)	0.6327 (4)	0.0272 (10)	
H4A	0.1189	0.0565	0.6519	0.033*	
H4B	0.1632	−0.0373	0.5911	0.033*	
Cd1	0.26771 (5)	0.14437 (4)	0.50053 (4)	0.03445 (11)	
Cd2	0.29638 (4)	0.51532 (3)	0.54349 (4)	0.02758 (10)	
I1	0.13000 (4)	0.34667 (3)	0.60022 (4)	0.02942 (10)	
I2	0.58302 (4)	0.43699 (3)	0.59181 (4)	0.02991 (10)	
I3	0.16270 (4)	0.56236 (3)	0.28416 (4)	0.03520 (10)	
I4	0.29511 (4)	0.70585 (3)	0.67547 (4)	0.03244 (10)	

### Atomic displacement parameters (Å^2^)

**Table:**

	*U*^11^	*U*^22^	*U*^33^	*U*^12^	*U*^13^	*U*^23^
C1	0.028 (3)	0.028 (3)	0.027 (3)	0.005 (2)	0.013 (2)	−0.003 (2)
C2	0.046 (4)	0.036 (3)	0.040 (3)	0.007 (3)	0.024 (3)	0.007 (3)
C3	0.069 (5)	0.047 (4)	0.033 (3)	0.005 (3)	0.024 (3)	0.004 (3)
C4	0.056 (4)	0.050 (4)	0.030 (3)	0.014 (3)	0.004 (3)	−0.005 (3)
C5	0.035 (3)	0.058 (4)	0.028 (3)	0.005 (3)	0.001 (3)	−0.009 (3)
C6	0.028 (3)	0.029 (3)	0.023 (3)	0.005 (2)	0.007 (2)	−0.003 (2)
C7	0.023 (3)	0.028 (3)	0.027 (3)	0.002 (2)	0.010 (2)	−0.004 (2)
C8	0.037 (3)	0.033 (3)	0.031 (3)	0.000 (2)	0.006 (3)	−0.009 (2)
C9	0.041 (4)	0.050 (4)	0.028 (3)	0.004 (3)	0.009 (3)	0.001 (3)
C10	0.048 (4)	0.035 (3)	0.037 (3)	0.006 (3)	0.024 (3)	0.006 (3)
C11	0.040 (3)	0.025 (3)	0.032 (3)	0.000 (2)	0.021 (3)	−0.002 (2)
C12	0.031 (3)	0.022 (3)	0.026 (3)	−0.001 (2)	0.012 (2)	−0.004 (2)
N1	0.019 (2)	0.029 (2)	0.035 (3)	−0.0024 (18)	0.008 (2)	−0.0027 (19)
N2	0.021 (2)	0.038 (3)	0.038 (3)	−0.0028 (19)	0.010 (2)	−0.001 (2)
N3	0.021 (2)	0.031 (2)	0.033 (3)	−0.0003 (18)	0.0095 (19)	0.002 (2)
N4	0.021 (2)	0.031 (2)	0.031 (2)	−0.0069 (18)	0.0106 (19)	−0.0069 (19)
Cd1	0.0276 (2)	0.0449 (2)	0.0285 (2)	−0.00507 (18)	0.00643 (17)	0.00808 (18)
Cd2	0.0274 (2)	0.0250 (2)	0.0318 (2)	−0.00032 (15)	0.01194 (17)	−0.00169 (16)
I1	0.02921 (19)	0.02459 (18)	0.0380 (2)	0.00129 (13)	0.01589 (16)	0.00353 (14)
I2	0.02389 (19)	0.02559 (18)	0.0414 (2)	−0.00116 (13)	0.01243 (15)	−0.00022 (15)
I3	0.0283 (2)	0.0457 (2)	0.0310 (2)	−0.00090 (16)	0.00928 (16)	0.00441 (16)
I4	0.02979 (19)	0.02561 (18)	0.0454 (2)	−0.00135 (14)	0.01721 (16)	−0.00706 (16)

### Geometric parameters (Å, º)

**Table:**

C1—N1	1.479 (7)	C10—C11	1.513 (9)
C1—C2	1.513 (8)	C10—H10A	0.9700
C1—C6	1.537 (7)	C10—H10B	0.9700
C1—H1C	0.9800	C11—C12	1.535 (8)
C2—C3	1.525 (9)	C11—H11A	0.9700
C2—H2C	0.9700	C11—H11B	0.9700
C2—H2D	0.9700	C12—N4	1.474 (7)
C3—C4	1.517 (11)	C12—H12A	0.9800
C3—H3C	0.9700	N1—Cd1	2.302 (5)
C3—H3D	0.9700	N1—H1A	0.9000
C4—C5	1.515 (10)	N1—H1B	0.9000
C4—H4C	0.9700	N2—Cd1	2.331 (5)
C4—H4D	0.9700	N2—H2A	0.9000
C5—C6	1.526 (7)	N2—H2B	0.9000
C5—H5A	0.9700	N3—Cd1	2.303 (4)
C5—H5B	0.9700	N3—H3A	0.9000
C6—N2	1.457 (7)	N3—H3B	0.9000
C6—H6A	0.9800	N4—Cd1	2.315 (5)
C7—N3	1.483 (7)	N4—H4A	0.9000
C7—C12	1.523 (7)	N4—H4B	0.9000
C7—C8	1.532 (8)	Cd1—I1	3.1646 (6)
C7—H7A	0.9800	Cd1—I2^i^	3.2353 (6)
C8—C9	1.517 (9)	Cd2—I4	2.7374 (5)
C8—H8A	0.9700	Cd2—I3	2.7609 (6)
C8—H8B	0.9700	Cd2—I1	2.8041 (5)
C9—C10	1.529 (9)	Cd2—I2	2.8070 (5)
C9—H9A	0.9700	I2—Cd1^ii^	3.2353 (6)
C9—H9B	0.9700		
			
N1—C1—C2	112.4 (5)	C10—C11—C12	111.8 (5)
N1—C1—C6	107.9 (4)	C10—C11—H11A	109.3
C2—C1—C6	113.4 (5)	C12—C11—H11A	109.3
N1—C1—H1C	107.6	C10—C11—H11B	109.3
C2—C1—H1C	107.6	C12—C11—H11B	109.3
C6—C1—H1C	107.6	H11A—C11—H11B	107.9
C1—C2—C3	113.3 (5)	N4—C12—C7	110.7 (4)
C1—C2—H2C	108.9	N4—C12—C11	112.1 (4)
C3—C2—H2C	108.9	C7—C12—C11	111.1 (4)
C1—C2—H2D	108.9	N4—C12—H12A	107.6
C3—C2—H2D	108.9	C7—C12—H12A	107.6
H2C—C2—H2D	107.7	C11—C12—H12A	107.6
C4—C3—C2	110.9 (5)	C1—N1—Cd1	110.3 (3)
C4—C3—H3C	109.5	C1—N1—H1A	109.6
C2—C3—H3C	109.5	Cd1—N1—H1A	109.6
C4—C3—H3D	109.5	C1—N1—H1B	109.6
C2—C3—H3D	109.5	Cd1—N1—H1B	109.6
H3C—C3—H3D	108.1	H1A—N1—H1B	108.1
C5—C4—C3	109.3 (5)	C6—N2—Cd1	108.6 (3)
C5—C4—H4C	109.8	C6—N2—H2A	110.0
C3—C4—H4C	109.8	Cd1—N2—H2A	110.0
C5—C4—H4D	109.8	C6—N2—H2B	110.0
C3—C4—H4D	109.8	Cd1—N2—H2B	110.0
H4C—C4—H4D	108.3	H2A—N2—H2B	108.3
C4—C5—C6	112.3 (5)	C7—N3—Cd1	109.8 (3)
C4—C5—H5A	109.2	C7—N3—H3A	109.7
C6—C5—H5A	109.2	Cd1—N3—H3A	109.7
C4—C5—H5B	109.2	C7—N3—H3B	109.7
C6—C5—H5B	109.2	Cd1—N3—H3B	109.7
H5A—C5—H5B	107.9	H3A—N3—H3B	108.2
N2—C6—C5	114.0 (5)	C12—N4—Cd1	109.8 (3)
N2—C6—C1	109.2 (4)	C12—N4—H4A	109.7
C5—C6—C1	110.7 (5)	Cd1—N4—H4A	109.7
N2—C6—H6A	107.6	C12—N4—H4B	109.7
C5—C6—H6A	107.6	Cd1—N4—H4B	109.7
C1—C6—H6A	107.6	H4A—N4—H4B	108.2
N3—C7—C12	110.1 (4)	N1—Cd1—N3	109.27 (16)
N3—C7—C8	112.3 (4)	N1—Cd1—N4	171.57 (16)
C12—C7—C8	109.8 (4)	N3—Cd1—N4	76.48 (15)
N3—C7—H7A	108.2	N1—Cd1—N2	75.63 (16)
C12—C7—H7A	108.2	N3—Cd1—N2	175.06 (17)
C8—C7—H7A	108.2	N4—Cd1—N2	98.71 (16)
C9—C8—C7	113.0 (5)	N1—Cd1—I1	95.78 (12)
C9—C8—H8A	109.0	N3—Cd1—I1	85.16 (12)
C7—C8—H8A	109.0	N4—Cd1—I1	90.79 (12)
C9—C8—H8B	109.0	N2—Cd1—I1	93.84 (13)
C7—C8—H8B	109.0	N1—Cd1—I2^i^	85.07 (12)
H8A—C8—H8B	107.8	N3—Cd1—I2^i^	92.92 (12)
C8—C9—C10	112.1 (5)	N4—Cd1—I2^i^	88.53 (12)
C8—C9—H9A	109.2	N2—Cd1—I2^i^	88.06 (13)
C10—C9—H9A	109.2	I1—Cd1—I2^i^	178.059 (17)
C8—C9—H9B	109.2	I4—Cd2—I3	106.571 (18)
C10—C9—H9B	109.2	I4—Cd2—I1	113.542 (17)
H9A—C9—H9B	107.9	I3—Cd2—I1	105.992 (17)
C11—C10—C9	110.4 (5)	I4—Cd2—I2	111.559 (17)
C11—C10—H10A	109.6	I3—Cd2—I2	110.876 (17)
C9—C10—H10A	109.6	I1—Cd2—I2	108.170 (16)
C11—C10—H10B	109.6	Cd2—I1—Cd1	98.931 (15)
C9—C10—H10B	109.6	Cd2—I2—Cd1^ii^	100.991 (14)
H10A—C10—H10B	108.1		
			
N1—C1—C2—C3	−171.1 (5)	C1—N1—Cd1—N4	34.1 (12)
C6—C1—C2—C3	−48.3 (7)	C1—N1—Cd1—N2	−14.4 (3)
C1—C2—C3—C4	53.4 (7)	C1—N1—Cd1—I1	−106.9 (3)
C2—C3—C4—C5	−58.4 (7)	C1—N1—Cd1—I2^i^	74.8 (3)
C3—C4—C5—C6	60.4 (7)	C7—N3—Cd1—N1	171.0 (3)
C4—C5—C6—N2	−178.3 (5)	C7—N3—Cd1—N4	−15.4 (3)
C4—C5—C6—C1	−54.7 (7)	C7—N3—Cd1—N2	−2 (2)
N1—C1—C6—N2	−60.4 (5)	C7—N3—Cd1—I1	76.6 (3)
C2—C1—C6—N2	174.4 (4)	C7—N3—Cd1—I2^i^	−103.2 (3)
N1—C1—C6—C5	173.3 (5)	C12—N4—Cd1—N1	120.8 (10)
C2—C1—C6—C5	48.1 (6)	C12—N4—Cd1—N3	−13.1 (3)
N3—C7—C8—C9	−176.9 (5)	C12—N4—Cd1—N2	168.1 (3)
C12—C7—C8—C9	−54.1 (6)	C12—N4—Cd1—I1	−97.9 (3)
C7—C8—C9—C10	53.6 (7)	C12—N4—Cd1—I2^i^	80.3 (3)
C8—C9—C10—C11	−53.5 (7)	C6—N2—Cd1—N1	−17.3 (3)
C9—C10—C11—C12	55.7 (6)	C6—N2—Cd1—N3	156 (2)
N3—C7—C12—N4	−55.2 (5)	C6—N2—Cd1—N4	169.0 (3)
C8—C7—C12—N4	−179.3 (4)	C6—N2—Cd1—I1	77.7 (3)
N3—C7—C12—C11	179.5 (4)	C6—N2—Cd1—I2^i^	−102.7 (3)
C8—C7—C12—C11	55.5 (6)	I4—Cd2—I1—Cd1	−161.301 (17)
C10—C11—C12—N4	177.6 (4)	I3—Cd2—I1—Cd1	82.052 (18)
C10—C11—C12—C7	−57.9 (6)	I2—Cd2—I1—Cd1	−36.908 (19)
C2—C1—N1—Cd1	168.5 (4)	N1—Cd1—I1—Cd2	−28.11 (11)
C6—C1—N1—Cd1	42.7 (4)	N3—Cd1—I1—Cd2	80.81 (11)
C5—C6—N2—Cd1	170.3 (4)	N4—Cd1—I1—Cd2	157.17 (11)
C1—C6—N2—Cd1	45.9 (5)	N2—Cd1—I1—Cd2	−104.04 (12)
C12—C7—N3—Cd1	41.4 (5)	I2^i^—Cd1—I1—Cd2	87.7 (5)
C8—C7—N3—Cd1	164.0 (3)	I4—Cd2—I2—Cd1^ii^	−57.90 (2)
C7—C12—N4—Cd1	39.5 (5)	I3—Cd2—I2—Cd1^ii^	60.712 (19)
C11—C12—N4—Cd1	164.2 (3)	I1—Cd2—I2—Cd1^ii^	176.530 (16)
C1—N1—Cd1—N3	166.2 (3)		

Symmetry codes: (i) −*x*+1, *y*−1/2, −*z*+1; (ii) −*x*+1, *y*+1/2, −*z*+1.

### Hydrogen-bond geometry (Å, º)

**Table:**

*D*—H···*A*	*D*—H	H···*A*	*D*···*A*	*D*—H···*A*
N1—H1*B*···I4^i^	0.90	2.84	3.723 (4)	167
N2—H2*B*···I4^iii^	0.90	2.81	3.681 (5)	162
N3—H3*A*···I3^i^	0.90	2.94	3.809 (4)	162
N4—H4*A*···I3^iii^	0.90	3.03	3.875 (5)	157

Symmetry codes: (i) −*x*+1, *y*−1/2, −*z*+1; (iii) −*x*, *y*−1/2, −*z*+1.
